# ﻿Four new species of genus *Acmella* W.T. Blanford, 1869 (Gastropoda, Assimineidae) from Southern Thailand

**DOI:** 10.3897/zookeys.1256.157322

**Published:** 2025-10-22

**Authors:** Kunya Seedee, Pongrat Dumrongrojwattana, Supattra Poeaim

**Affiliations:** 1 Department of Biology, School of Science, King Mongkut’s Institute of Technology Ladkrabang, Bangkok 10520, Thailand School of Science, King Mongkut’s Institute of Technology Ladkrabang Bangkok Thailand; 2 Department of Biology, Faculty of Science, Burapha University, Bangsaen, Chonburi 20131, Thailand Burapha University Chonburi Thailand

**Keywords:** Cave-dwelling gastropods, limestone habitat, microsnails

## Abstract

This study explores the diversity of microsnails inhabiting limestone caves in Southern Thailand. It describes four new species of the genus *Acmella* W.T. Blanford, 1869 (Gastropoda, Assimineidae): *Acmella krueangensis***sp. nov.** from Ranong Province, *A. thamsingensis***sp. nov.** and *A. changphueakensis***sp. nov.** from Chumphon Province, and *A. kanchanaditensis***sp. nov.** from Surat Thani Province. These species are distinguished by shell morphology, particularly the number of striae on the last whorl and the protoconch sculpture. Phylogenetic analyses based on mitochondrial cytochrome c oxidase subunit I (*COI*) gene sequences further support their distinctiveness and clarify their taxonomic placement. The results enhance the understanding of *Acmella* diversity in Thailand and shed light on biogeographical patterns found in this genus and the adaptations needed to survive under the conditions of cave systems.

## ﻿Introduction

Microsnails of the family Assimineidae are predominantly distributed in mangrove habitats throughout Southeast Asia. However, the genus *Acmella* W.T. Blanford, 1879, is an exception, exhibiting a unique adaptation to limestone hills and karst formations with a broad distribution range across Asia. Members of *Acmella* are characterized by their minute, narrowly conical, white shells, typically measuring 1–2 mm in height. The shell comprises 4.5–5.0 whorls with a distinctly deep suture. The aperture is thin and subovate, and the umbilicus is open and narrow. The shell surface is marked by radial and axial striations, producing a distinctive sculptural pattern (Vermeulen et al. 2015; Das et al. 2021).

Morphological studies have documented a total of 34 species of *Acmella* across Asia, including 12 species from Malaysia (von Möllendorff 1887; Vermeulen and Junau 2007; Vermeulen et al. 2015; Phung et al. 2017, 2018; bin Marzuki et al. 2021; Foon and bin Marzuki 2022; Vermeulen and Liew 2022), 11 from Indonesia (Tapparone Canefri 1883; Boettger 1891; Van Benthem Jutting 1958, 1963; Maassen 2000; Vermeulen et al. 2015), eight from the Philippines (von Möllendorff 1893; Quadras and von Möllendorff 1895; Vermeulen et al. 2015; Auffenberg and Páll-Gergely 2020), four from India (Benson 1853; Nevill 1878; Godwin-Austen 1895; Das et al. 2021; Chen and Páll-Gergely 2023), one from Myanmar (Theobald and Stoliczka 1872), and one from Vietnam (Vermeulen et al. 2019). In Thailand, four undescribed species of *Acmella* have been recorded (Wangkhiri et al. 2018; Suksai and Dumrongrojwattana 2022). This diversity highlights the genus’s ecological specialization and biogeographic importance in the region.

Notably, three microsnail species from the Philippines, *Georissa regularis* Quadras & Möllendorff, 1895, *G. subglabrata* Möllendorff, 1887, and *G. turritella* Möllendorff, 1893, have been reclassified under *Acmella* based on distinctive differences in shell morphology, including overall shell shape, number of whorls, and aperture characteristics (Auffenberg and Páll-Gergely 2020). *Cyclostoma tersum* W.H. Benson, 1853, was designated as type species (by monotypy) when the genus was established by Blanford in 1869. Key shell features include transverse sculpture elements on the teleoconch, a non-circular aperture, and an unexpanded peristome. *Acmella* differs from the closely related *Anaglyphula* B. Rensch, 1932 by lacking an internal constriction near the aperture (Chen and Páll-Gergely 2023).

Currently, two species of *Acmella* from Sabah, Malaysian Borneo, *A. cyrtoglyphe* Vermeulen, Liew & Schilthuizen, 2015 and *A. polita* Möllendorff, 1887, are represented in the National Center for Biotechnology Information (NCBI) database. These species were identified using DNA barcoding based on several genetic markers, including 16S ribosomal RNA (16S rRNA), 18S ribosomal RNA (18S rRNA), 28S ribosomal RNA (28S rRNA), cytochrome c oxidase subunit I (*COI*), and histone H3 (H3) (Hendriks 2020). In Thailand, four species of *Acmella* have been recorded solely based on morphological characteristics, with shell sculpture, particularly on the last whorl, serving as the principal diagnostic trait.

So, the present study aims to investigate the diversity of microsnails in the limestone caves of Southern Thailand, a region recognized for its high biodiversity. It further seeks to provide the first molecular identification of *Acmella* species from Thailand using DNA barcoding, focusing primarily on the mitochondrial *COI* gene.

## ﻿Materials and methods

### ﻿Specimen sampling

**Figure 1.**
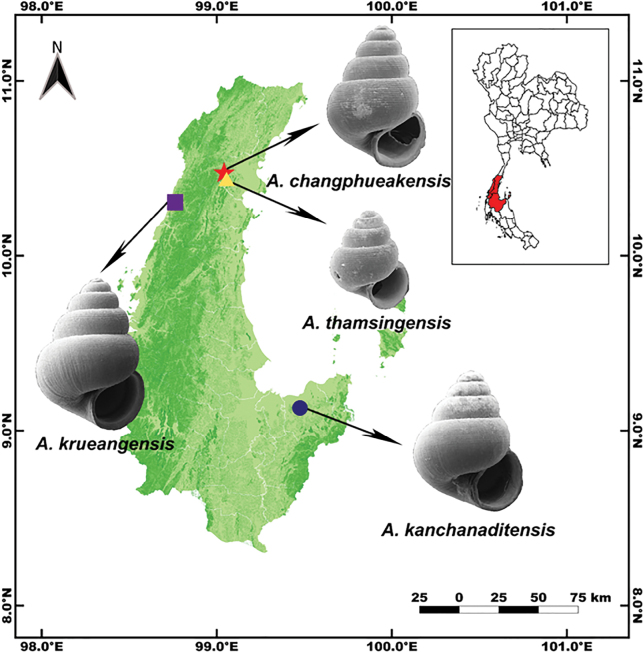
Type localities of the four new *Acmella* species described from Southern Thailand: *A. krueangensis* sp. nov. (purple), Ranong Province; *A. thamsingensis* sp. nov. (yellow), Chumphon Province; *A. changphueakensis* sp. nov. (red), Chumphon Province; and *A. kanchanaditensis* sp. nov. (blue), Surat Thani Province.

Field surveys were conducted at four limestone cave sites in Southern Thailand: Phra Krueang Cave, Ranong Province (10.3265, 98.7646); Wat Tham Sing, Chumphon Province (10.4256, 99.0600); Samnaksong Tham Chang Phueak, Chumphon Province (10.4463, 99.0349); and Wat Pa Kanchanadit, Surat Thani Province (9.1426, 99.4708) (Fig. 1). Specimens were collected manually from within the caves, including individuals found attached to rock crevices and shells embedded in soil containing shell debris. All specimens were found exclusively in aphotic cave environments, particularly in moist microhabitats where water seeped through cave walls, stalactites, and stalagmites. Specimens were collected at depths or distances ranging from 15 to 30 m from the cave entrances, with a mean of 21.25 ± 6.29 m. In addition to live specimens, fossilized shells were occasionally observed in areas that were likely water-saturated in the past. Their coloration and small size make them difficult to detect even when alive, as their shells blend into the surrounding substrate like grains of sand. None of these species were found outside of the cave environments. All collected specimens were preserved in 50% ethanol to ensure tissue integrity for subsequent morphological and molecular analyses. Sample processing and preservation were performed at the Department of Biology, School of Science, King Mongkut’s Institute of Technology Ladkrabang (KMITL), and Burapha University (BUU).

### ﻿Morphological examination

Morphological examinations were conducted using a stereomicroscope. Soft tissues were carefully separated from the shells and preserved in 50% ethanol for subsequent molecular analysis. Shells were cleaned using hydrogen peroxide and rinsed with distilled water to remove debris and organic residues. Specimens were photographed using a digital camera to document shell morphology. Shell measurements, including shell height (SH), shell width (SW), aperture height (AH), and aperture width (AW), were taken using ImageJ software (Schneider et al. 2012) to assess and compare shell shape among specimens. Scanning electron microscopy (SEM) was employed to examine protoconch sculpture, sculpture on the last whorl, and operculum morphology, following the protocols of Panha and Burch (2005). The sculpture on the last whorl was categorized into two major types following Vermeulen et al. (2015): (1) radial sculpture predominant, and (2) spiral sculpture predominant, radial and spiral sculpture approximately equally developed, or sculpture virtually absent. Operculum morphology was examined and described based on the diagnostic framework provided by Vermeulen et al. (2015), focusing on characteristics such as thinness, transparency, paucispiral coiling, and chitinous composition, which were used as taxonomic criteria for species identification within *Acmella*.

### ﻿Molecular analysis

Genomic DNA was extracted from tissues of four morphologically distinct *Acmella* species (two individuals per species) using the GF-1 Tissue DNA Extraction Kit (Vivantis Technologies, Malaysia), following the manufacturer’s protocol. For each specimen, tissue was obtained by drilling a small hole (approximately 0.4 × 0.4 mm) on the dorsal side of the last whorl. An acupuncture needle (0.12 × 0.13 mm) was used to carefully extract the tissue for DNA analysis. The shell was preserved intact for subsequent morphological examination. A fragment of the mitochondrial cytochrome c oxidase subunit I (*COI*) gene was amplified using the universal primers LCO1490 (5′–GGTCAACAAATCATAAAGATATTG–3′) and HCO2198 (5′–TAAACT TCAGGGTGACCAAAAAATCA–3′) (Folmer et al. 1994). Polymerase chain reaction (PCR) was performed in a 25 µL reaction volume consisting of 2.5 µL of 10 × PCR buffer, 1 µL of 25 mM MgCl_2_, 1 µL of 1.25 mM dNTPs, 1 µL of each primer (10 pmol), 0.25 µL of Taq DNA polymerase (New England Biolabs), 1 µL of genomic DNA template, and 17.25 µL of nuclease-free water. The PCR cycling protocol included an initial denaturation at 95 °C for 5 min, followed by 40 cycles of denaturation at 95 °C for 15 s, annealing at 50 °C for 30 s, and extension at 72 °C for 1 min, with a final extension at 72 °C for 5 min (Khalik et al. 2018). Amplification success was confirmed by electrophoresis on 1.5% agarose gels stained with ethidium bromide. PCR products were purified and sequenced using Barcode Indexed Tag Sequencing (BITseq). The resulting *COI* gene sequences were edited and aligned using MEGA 11 (Tamura et al. 2021). Sequence identities were assessed using BLAST searches against the NCBI GenBank database, focusing on available *Acmella* and *Georissa* species sequences. The best-fitting nucleotide substitution model, the General Time Reversible model (GTR), was selected using jModelTest v. 1.4.4 (Darriba et al. 2012), based on the Bayesian Information Criterion (BIC). Phylogenetic relationships were reconstructed using Bayesian Inference (BI) in MrBayes v. 3.2.7 (Ronquist et al. 2012), with 1,000,000 generations and four Markov chains. Trees are sampled every 100 generations, and the first 25% are discarded as burn-in.

## ﻿Results

### ﻿Morphological studies

Four new species of *Acmella* were discovered during faunal surveys of limestone cave systems in Southern Thailand, specifically in Phra Krueang Cave (Ranong Province), Wat Tham Sing and Samnaksong Tham Chang Phueak (Chumphon Province), and Wat Pa Kanchanadit (Surat Thani Province) (Fig. 1).

**Figure 2.**
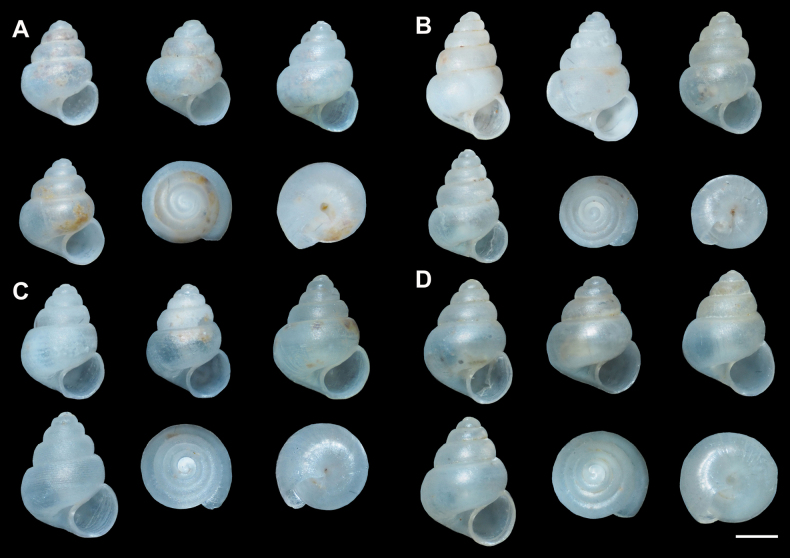
Shell morphology of the four new *Acmella* species described from Southern Thailand, based on paratype specimens (4 samples per species): A. *A. krueangensis* sp. nov., ZRCBUU 0988, PV1579890; B. *A. thamsingensis* sp. nov., ZRCBUU 0990, PV157992; C. *A. changphueakensis* sp. nov., ZRCBUU 0992, PV157994; D. *A. kanchanaditensis* sp. nov., ZRCBUU 0994, PV157996. Digital microscope images of paratypes illustrating shell shape variation are shown. Scale bars: 0.5 mm (A–D).

The morphological analysis of the holotypes of *Acmella* species revealed that SH ranged from 1.01 to 1.51 mm (mean 1.33 ± 0.22 mm), SW ranged from 0.81 to 1.10 mm (mean 0.99 ± 0.14 mm), AH ranged from 0.41 to 0.58 mm (mean 0.52 ± 0.08 mm), and AW ranged from 0.42 to 0.53 mm (mean 0.50 ± 0.05 mm). A relatively narrow range of shell dimensions was observed among species. The analysis of additional specimens (paratypes) revealed significant variation in shell measurements. *Acmella krueangensis* sp. nov. exhibited the greatest size variation, with SH ranging from 0.97 to 1.55 mm (mean 1.37 ± 0.23 mm) and SW from 0.68 to 1.14 mm (mean 1.01 ± 0.18 mm), representing both the smallest and largest individuals observed. *Acmella thamsingensis* sp. nov. had SH ranging from 1.37 to 1.43 mm (mean 1.40 ± 0.03 mm) and SW from 0.93 to 1.03 mm (mean 0.98 ± 0.05 mm). *Acmella changphueakensis* sp. nov. exhibited SH between 1.33 and 1.49 mm (mean 1.38 ± 0.08 mm) and SW between 0.95 and 1.09 mm (mean 1.01 ± 0.07 mm). *Acmella kanchanaditensis* sp. nov. showed the largest average shell height, with SH ranging from 1.39 to 1.52 mm (mean 1.47 ± 0.08 mm), and the most consistent shell width, from 1.07 to 1.10 mm (mean 1.08 ± 0.01 mm). Digital microscope images of paratypes illustrating intraspecific shell shape variation are shown. Four paratype specimens for each species are presented in Fig. 2A–D.

Morphological analysis of the four species of *Acmella* revealed distinct shell sculptures that can be used as diagnostic features for species identification. *Acmella krueangensis* sp. nov. exhibits a reticulate pattern with clearly defined radial and spiral ridges, creating a mesh-like appearance (Fig. 4). *Acmella thamsingensis* sp. nov. shows pronounced spiral ridges with radial sculptures that are most prominent on the upper whorls and gradually fade toward the lower whorls (Fig. 5). *Acmella changphueakensis* sp. nov. is characterized by dominant spiral ridges with faint radial lines, which are nearly invisible in some areas (Fig. 6). *Acmella kanchanaditensis* sp. nov. shares a similar sculpture to *A. changphueakensis* sp. nov., but it has a higher density of fine spiral ridges and narrower spacing between them (Fig. 7). While these species share similar overall shell morphology, including shape, coloration, and operculum structure, the shell sculpture provides the most reliable distinguishing feature among them.

### ﻿Phylogenetic analysis

The phylogenetic analysis was based on eight newly generated *COI* sequences from four newly described *Acmella* species: *A. krueangensis* sp. nov., *A. thamsingensis* sp. nov., *A. changphueakensis* sp. nov., and *A. kanchanaditensis* sp. nov. (two individuals per species). The sequences were approximately 700 bp in length, as estimated by agarose gel electrophoresis, and were subsequently trimmed to 537 bp for analysis. All sequences were deposited in GenBank (Table 1). Additional *COI* sequences of two previously described species, *A. polita* and *A. cyrtoglyphe* from Borneo, were included, along with four species of *Georissa* W.T. Blanford, 1864 (*G. quinquelirata* Klongkaew, Poeaim & Dumrongrojwattana, 2024, *G. digitinota* Klongkaew, Poeaim & Dumrongrojwattana, 2024, *G. sagitta* Klongkaew, Poeaim & Dumrongrojwattana, 2024, and *G. kohsichangensis* Klongkaew, Poeaim & Dumrongrojwattana, 2024) as outgroups. Sequences were aligned using ClustalW with default parameters and manually adjusted in MEGA11. Phylogenetic reconstruction was conducted using MrBayes v. 3.2.7 under the General Time Reversible model with a gamma distribution and a proportion of invariable sites.

**Table 1.** List of *Acmella* and *Georissa* species, specimen catalog numbers, GenBank accession numbers, and references included in the phylogenetic analysis.

Species	Specimen Catalog Numbers	Accession Numbers	References
*A. polita*	RMNH.5005040.01	MK851191	Hendriks 2020
*A. cyrtoglyphe*	BOR/MOL7316.01	MK851202	
*G. quinquelirata*	ZRCBUU 0900	PP844569	Klongklaew et al. 2024
*G. digitinota*	ZRCBUU 0906	PP844575	
*G. sagitta*	ZRCBUU 0904	PP844573	
*G. kohsichangensis*	ZRCBUU 0902	PP844571	
*A. krueangensis* sp. nov.	ZRCBUU 0987	PV157989	This study
*A. krueangensis* sp. nov.	ZRCBUU 0988	PV157990	
*A. thamsingensis* sp. nov.	ZRCBUU 0989	PV157991	
*A. thamsingensis* sp. nov.	ZRCBUU 0990	PV157992	
*A. changphueakensis* sp. nov.	ZRCBUU 0991	PV157993	
*A. changphueakensis* sp. nov.	ZRCBUU 0992	PV157994	
*A. kanchanaditensis* sp. nov.	ZRCBUU 0993	PV157995	
*A. kanchanaditensis* sp. nov.	ZRCBUU 0994	PV157996	

**Figure 3.**
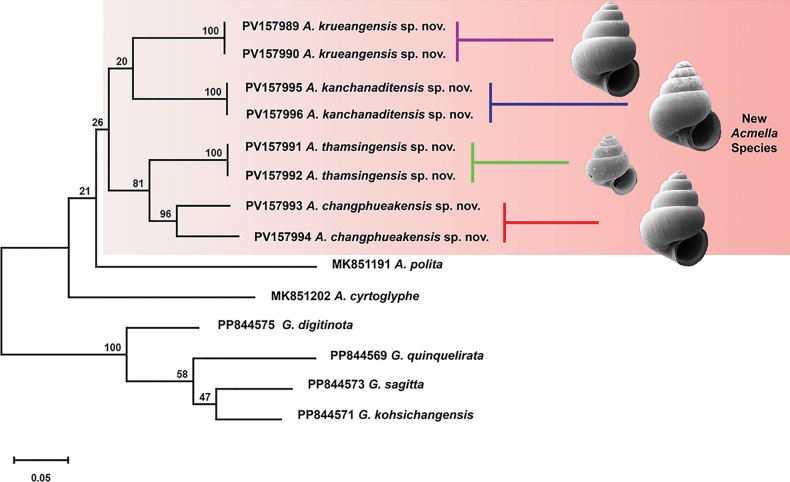
Phylogenetic tree of *Acmella* species based on the *COI* gene. The tree was constructed using the Bayesian-inference method with the General Time Reversible (GTR) + G + I model. The posterior probability is displayed at each node in the nucleotide sequence.

The Bayesian tree recovered the four new Thai *Acmella* species as a well-supported monophyletic clade, with all major nodes receiving strong support (posterior probabilities > 0.95). Within this clade, *A. thamsingensis* sp. nov. and *A. changphueakensis* sp. nov. formed a strongly supported sister group, whereas *A. krueangensis* sp. nov. and *A. kanchanaditensis* sp. nov. diverged independently, reflecting their distinct shell morphologies. The *COI* gene analysis grouped Thai specimens and *A. polita* and *A. cyrtoglyphe* from Borneo into a single unresolved clade (Fig. 3), lacking sufficient resolution to clarify their evolutionary relationships.

**Figure 4.**
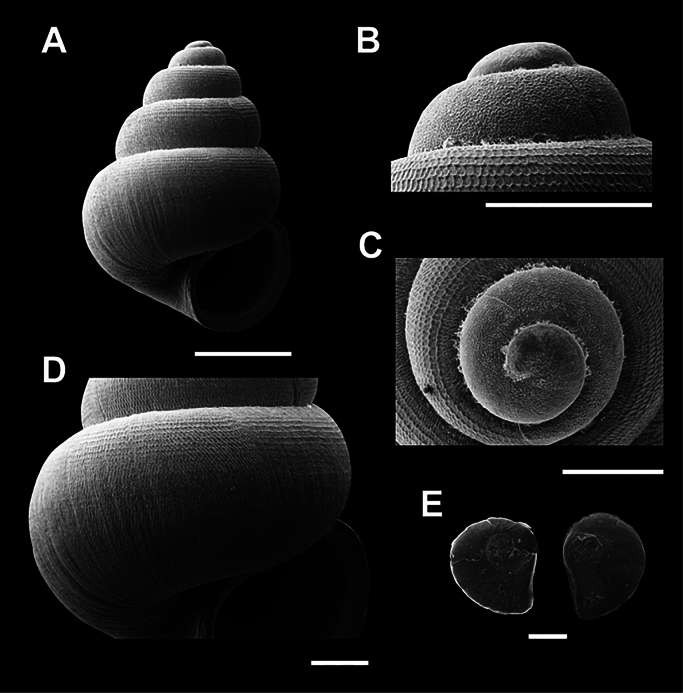
Scanning electron micrographs (SEM) of *Acmella krueangensis* sp. nov. (A–E) various shell views. A. Overall shell view; B, C. Protoconch and sculpture details; D. Last whorl sculpture; E. Operculum. Scale bars: 0.5 mm (A, B); 0.1 mm (C–E).

**Figure 5.**
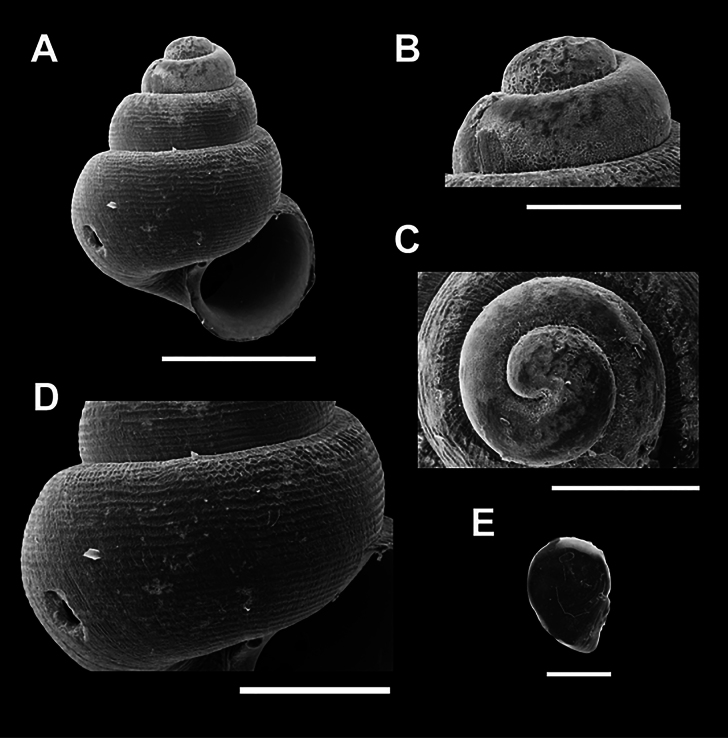
Scanning electron micrographs (SEM) of *Acmella thamsingensis* sp. nov. (A–E) various shell views. A. Overall shell view; B, C. Protoconch and sculpture details; D. Last whorl sculpture; E. Operculum. Scale bars: 0.5 mm (A, B); 0.1 mm (C–E).

**Figure 6.**
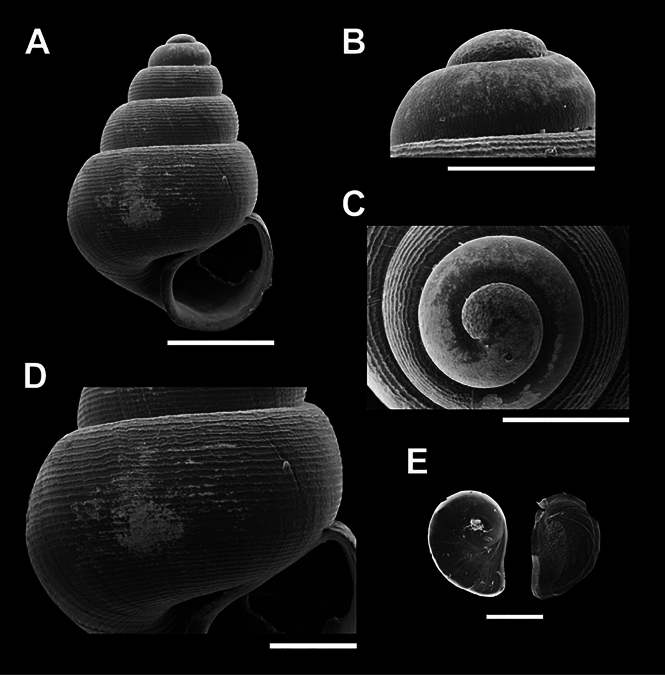
Scanning electron micrographs (SEM) of *Acmella changphueakensis* sp. nov. (A–E) various shell views. A. Overall shell view; B, C. Protoconch and sculpture details; D. Last whorl sculpture; E. Operculum. Scale bars: 0.5 mm (A, B); 0.1 mm (C–E).

**Figure 7.**
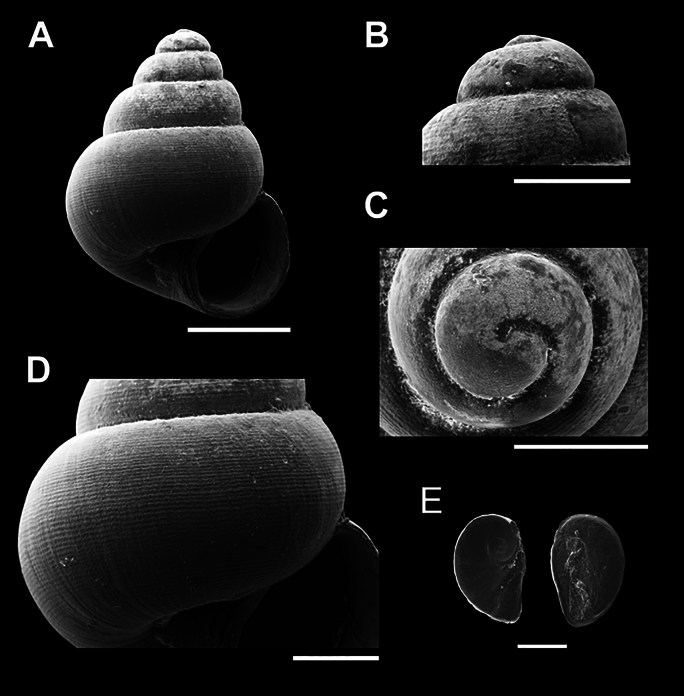
Scanning electron micrographs (SEM) of *Acmella kanchanaditensis* sp. nov. (A–E) various shell views. A. Overall shell view; B, C. Protoconch and sculpture details; D. Last whorl sculpture; E. Operculum. Scale bars: 0.5 mm (A, B); 0.1 mm (C–E).

### ﻿Systematics

#### ﻿Family Assimineidae H. Adams & A. Adams, 1856

**Subfamily Ekadantinae Thiele, 1929**

##### ﻿Genus Acmella

Taxon classificationAnimaliaLittorinimorphaAssimineidae

W.T. Blanford, 1869

F1317B8D-5AA1-57C5-A6BB-DC1A0E6950A0

###### Type species.

*Cyclostoma tersum* Benson, 1853.

##### ﻿ Acmella krueangensis

Taxon classificationAnimaliaLittorinimorphaAssimineidae

Seedee, Dumrongrojwattana & Poeaim sp. nov.

CD7E2B23-C490-5593-A0E0-804970919958

https://zoobank.org/F6C58A3A-78C2-4A2A-8FAB-24323F5F49BB

Fig. 4

###### Type material.

***Holotype***: Thailand • Phra Krueang Cave, Bang Bon Subdistrict, Kra Buri District, Ranong Province; 10.3265, 98.7646; November 2023; Kunya Seedee; deposited in the Malacological Collection; catalog no. ZRCBUU 0987; GenBank accession number: PV157989, SH = 1.51 mm, SW = 1.09 mm, AH = 0.58 mm, AW = 0.53 mm (Fig. 4A–E, Table 2). ***Paratype***: Thailand • 6 shells; same locality data as holotype; November 2023; deposited in the same institution as the holotype; catalog no. ZRCBUU 0988; GenBank accession number: PV157990, SH = 0.97–1.55 mm (1.37 ± 0.23 mm), SW = 0.68–1.14 mm (1.01 ± 0.18 mm), AH = 0.39–0.58 mm (0.57 ± 0.07 mm), AW = 0.45–0.56 mm (0.52 ± 0.08 mm).

**Table 2.** Holotype size measurements of *Acmella* in this study (in mm).

Species	Numbers	SH	SW	AH	AW
*A. krueangensis* sp. nov.	ZRCBUU 0987	1.51	1.09	0.58	0.53
*A. thamsingensis* sp. nov.	ZRCBUU 0989	1.01	0.81	0.41	0.42
*A. changphueakensis* sp. nov.	ZRCBUU 0991	1.37	0.96	0.52	0.53
*A. kanchanaditensis* sp. nov.	ZRCBUU 0993	1.41	1.10	0.58	0.51

###### Diagnosis.

Shell conical, white, translucent; 4½–5 convex whorls with a deep suture. Aperture obliquely oval. The last whorl sculpture is characterized by a distinct reticulated (mesh-like) pattern. Umbilicus narrow and open.

###### Description.

Shell conical, white, and translucent, with a rounded apex. Whorls convex, with a deep suture, totaling 4½–5 whorls. The aperture is obliquely oval, with a thin peristome and a concavity adjoining the upper body. Umbilicus open, narrow, and shallow (Fig. 4A). Protoconch with scattered malleated sculptures extending to the second whorl (Fig. 4B, C). The last whorl has a well-defined reticulated sculpture, formed by conspicuous radial and spiral elements, with unevenly tapered spiral lines (Fig. 4D). Operculum is thin, transparent, paucispiral, and chitinous (Fig. 4E).

###### Differential diagnosis.

*Acmella krueangensis* sp. nov. closely resembles *A. cyrtoglyphe* in general shell sculpture, particularly the presence of both radial and spiral elements. However, *A. krueangensis* sp. nov. differs by exhibiting more pronounced and evenly spaced radial ribs and a clearer reticulate (net-like) pattern formed by the intersection of radial and spiral elements. In contrast, the other three species possess sculpture dominated by spiral elements with less prominent or absent radial components, resulting in a predominantly spiral pattern rather than a net-like appearance.

###### Etymology.

The specific epithet *krueangensis* refers to Phra Krueang Cave, the type locality where the new species was discovered.

###### Habitat and distribution.

Currently known only from the type locality, Phra Krueang Cave, Ranong Province, Thailand.

###### Ecology.

This species inhabits dark, moist cave environments characterized by stalactites and stalagmites without evidence of surface water seepage.

##### ﻿ Acmella thamsingensis

Taxon classificationAnimaliaLittorinimorphaAssimineidae

Seedee, Dumrongrojwattana & Poeaim sp. nov.

5FE63AB3-94CA-5B42-9EDC-79D2156FBB70

https://zoobank.org/E7DACE7F-DD7E-4BE2-8C56-432220B03FF5

Fig. 5

###### Type material.

***Holotype***: Thailand • WatTham Sing, Tham Sing Subdistrict, Mueang District, Chumphon Province; 10.4256, 99.0600; November 2023; Kunya Seedee; deposited in the Malacological Collection; catalog no. ZRCBUU 0989; GenBank accession number: PV157991, SH = 1.01 mm, SW = 0.81 mm, AH = 0.41 mm, AW = 0.42 mm (Fig. 5A–E, Table 2). ***Paratype***: Thailand • 4 shells; same locality data as holotype; November 2023; deposited in the same institution as the holotype; catalog no. ZRCBUU 0990; GenBank accession number: PV157992, SH = 1.37–1.43 mm (1.40 ± 0.03 mm), SW = 0.93–1.03 mm (0.98 ± 0.05 mm), AH = 0.46–0.57 mm (0.49 ± 0.05 mm), AW = 0.37–0.49 mm (0.44 ± 0.06 mm).

###### Diagnosis.

Shell shape conical, white; suture visible; whorls 4–6. Aperture oblique oval. The last whorl sculpture consists of a lattice pattern on the upper part of the whorls. Umbilicus open and narrow.

###### Description.

Shell conical, white, translucent; rounded apex; whorls convex on the side up to a deep suture; 4¼–6 whorls. The aperture is oblique oval, with a wall next to the upper body, a concave part following the shell shape, and a thin peristome. The umbilicus is open, narrow, and superficial (Fig. 5A). Protoconch is a rough sculpture scattered throughout the area up to the second whorl (Fig. 5B, C). The last whorl sculpture is a spiral low convex sculpture with 23–26 rows, wide, conspicuous, and regularly spaced with a narrow groove in the wide rows. Radial sculpture tapered in the upper part of the whorl (Fig. 5D). Operculum paucispiral, thin, transparent, and composed of chitin (Fig. 5E).

###### Differential diagnosis.

The spiral sculpture of *A. thamsingensis* sp. nov. is more prominent than that of *A. cyrtoglyphe*, where it is almost absent. The radial sculpture of *A. thamsingensis* sp. nov. is limited to the upper part of the whorl, unlike *A. cyrtoglyphe*, which exhibits a more distinct and complete radial sculpture. Compared to *A. krueangensis* sp. nov., which has evenly distributed radial sculpture across the shell, *A. thamsingensis* sp. nov. bears radial elements only on the upper portion of the last whorl. In contrast, *A. changphueakensis* sp. nov. and *A. kanchanaditensis* sp. nov. lack radial sculpture.

###### Etymology.

The specific name *thamsingensis* refers to Wat Tham Sing, where the species was discovered.

###### Habitat and distribution.

This species is only found at the study site.

###### Ecology.

The new species was discovered in aphotic cave zones, inhabiting moist microhabitats where water percolates through the cave walls, approximately 20 m from the entrance.

##### ﻿ Acmella changphueakensis

Taxon classificationAnimaliaLittorinimorphaAssimineidae

Seedee, Dumrongrojwattana & Poeaim sp. nov.

45DC3B8D-7FE9-520C-A29B-8BA14E1E6A7A

https://zoobank.org/334BE3B4-63B6-429F-A1F4-343BD9A03E73

Fig. 6

###### Type material.

***Holotype***: Thailand • Samnaksong Tham Chang Phueak, Ban Na Subdistrict, Mueang District, Chumphon Province; 10.4463, 99.0349; November 2023; Kunya Seedee; Deposited in the Malacological Collection; catalog no. ZRCBUU 0991; GenBank accession number: PV157993, SH = 1.37 mm, SW = 0.96 mm, AH = 0.52 mm, AW = 0.53 mm (Fig. 6A–E, Table 2). ***Paratype***: Thailand • 4 shells; same locality data as holotype; November 2023; Deposited in the same institution as the holotype; catalog no. ZRCBUU 0992; GenBank accession number: PV157994, SH = 1.33–1.49 mm (1.38 ± 0.08 mm), SW = 0.95–1.09 mm (1.01 ± 0.07 mm), AH = 0.51–0.57 mm (0.54 ± 0.03 mm), AW = 0.48–0.54 mm (0.50 ± 0.03 mm).

###### Diagnosis.

Shell conical, white; a visible suture; 5–5½ whorls. Aperture oblique and oval. The last whorl sculpture has a prominently spiraled, low convex pattern. Umbilicus open and narrow.

###### Description.

Shell conical, white, translucent; apex rounded; whorls convex up to deep suture; 5–5½ whorls. The aperture is oblique oval, with a wall adjacent to the upper body, a concave portion following the shell shape, and a thin peristome. Umbilicus open, narrow, and nearly closed (Fig. 6A). Protoconch sculpture consisting of malleation, scattered throughout the area up to the second whorl (Fig. 6B, C). The last whorl sculpture consists of a spiral low convex pattern with 30–32, conspicuous, regularly spaced rows, each with a narrow groove. The radial sculpture is almost absent and irregularly spaced (Fig. 6D). Operculum paucispiral, thin, transparent, and composed of chitin (Fig. 6E).

###### Differential diagnosis.

*Acmella changphueakensis* sp. nov. resembles *A. minutissima* Maassen, 2000 in having a similar sculpture pattern on the last whorl. However, it can be distinguished by its higher number of spiral rows and greater number of whorls (five), whereas *A. minutissima* has only four. Among the newly described species, *A. changphueakensis* sp. nov. differs from *A. krueangensis* sp. nov. and *A. thamsingensis* sp. nov. by its faint radial sculpture, which is well developed in the latter two species. It shares a similar spiral sculpture with *A. kanchanaditensis* sp. nov., but it differs in having more widely spaced spiral rows.

###### Etymology.

The name *changphueakensis* is derived from Samnaksong Tham Chang Phueak, where this species was discovered.

###### Habitat and distribution.

This species is known only from the study site.

###### Ecology.

The new species inhabits cave environments where no light penetrates, specifically in areas where water seeps through the cave walls.

##### ﻿ Acmella kanchanaditensis

Taxon classificationAnimaliaLittorinimorphaAssimineidae

Seedee, Dumrongrojwattana & Poeaim sp. nov.

A47CDAA8-EB5B-5380-A34C-AF9CAE148709

https://zoobank.org/4345A7C6-45F0-48DF-BFC8-5E0FE5B628F5

Fig. 7

###### Type material.

***Holotype***: Thailand • Wat Pa Kanchanadit, Kadae Subdistrict, Kanchanadit District, Surat Thani Province; 9.1426, 99.4708; November 2023; Kunya Seedee; deposited in the Malacological Collection; catalog no. ZRCBUU 0993; GenBank accession number: PV157995, SH = 1.41 mm, SW = 1.10 mm, AH = 0.58 mm, AW = 0.51 mm (Fig. 7A-E, Table 2). ***Paratype***: Thailand • 4 shells; same locality data as holotype; November 2023; deposited in the same institution as the holotype; catalog no. ZRCBUU 0994; GenBank accession number: PV157996, SH = 1.39–1.52 mm (1.47 ± 0.08 mm), SW = 1.07–1.10 mm (1.08 ± 0.01 mm), AH = 0.51–0.60 mm (0.57 ± 0.04 mm), AW = 0.48–0.50 mm (0.49 ± 0.01 mm).

###### Diagnosis.

Shell conical, white; a visible suture; 4¾–5 whorls. Aperture oblique and oval. The last whorl sculpture has a relief spiral pattern, thin lines and is barely visible. Umbilicus open and narrow.

###### Description.

Shell conical, white, translucent; apex rounded; whorls convex up to deep suture; 4¾–5 whorls. Aperture oblique oval, with a wall adjacent to the upper body, a concave part following the shell shape, and a thin peristome. Umbilicus open, narrow, and superficial (Fig. 7A). Protoconch sculpture rough and spread across the area (Fig. 7B, C). Last whorl sculpture consists of 48 spiral rows with a shallow groove between them. Radial sculpture thin and barely visible (Fig. 7D). Operculum paucispiral, thin, transparent, composed of chitin (Fig. 7E).

###### Differential diagnosis.

*Acmella kanchanaditensis* sp. nov. can be distinguished from *A. caelata* Vermeulen & Junau, 2007 by its almost invisible radial sculpture. In contrast, in *A. caelata*, the radial sculpture remains faintly visible in certain areas. Additionally, *A. kanchanaditensis* sp. nov. has a higher number of whorls. This new species also closely resembles *A. changphueakensis* sp. nov. in having spiral sculpture, but the spiral rows in *A. kanchanaditensis* sp. nov. are thinner and more widely spaced. In contrast, *A. krueangensis* sp. nov. and *A. thamsingensis* sp. nov. exhibit well-developed radial sculpture, which is lacking in the present species.

###### Etymology.

The specific name *kanchanaditensis* is derived from Wat Pa Kanchanadit, where this species was discovered.

###### Habitat and distribution.

This species is known only from the study site.

###### Ecology.

The new species is found in caves where no light penetrates, specifically in areas where water seeps through stalactites and stalagmites.

## ﻿Discussion

The discovery of four new species of *Acmella* (*A. krueangensis* sp. nov., *A. thamsingensis* sp. nov., *A. changphueakensis* sp. nov., and *A. kanchanaditensis* sp. nov.) from aphotic cave systems in Southern Thailand significantly advances our understanding of the genus biodiversity. This study represents only the second confirmed report of *Acmella* inhabiting subterranean habitats, following the record of *A. tersa* from Meghalaya, India (Das et al. 2021). In contrast, *Acmella* species from Malaysia and Vietnam have only been recorded from above-ground leaf litter in limestone karst forests (Vermeulen et al. 2019, Vermeulen and Liew 2022; Foon and bin Marzuki 2022). The occurrence of *Acmella* in aphotic cave systems of the Sundaland region highlights the possibility of cave adaptation in the genus, a topic that warrants further investigation. Comparable research on cave adaptation and evolution in *Georissa* spp. (Khalik et al. 2020; Prieto et al. 2022) offers useful frameworks for future evolutionary and ecological studies. These newly described species exhibit distinct shell morphologies, particularly in the sculpture patterns of the last whorl, which serve as important diagnostic characters. For instance, *A. krueangensis* sp. nov. is characterized by a mesh-like sculpture, a pattern previously reported in an *Acmella* from Thailand by Wangkhiri et al. (2018). Although specimens in that study were collected from the same locality, the investigation focused solely on morphological characteristics. The present study builds upon these earlier findings by integrating morphological and molecular data, thus providing a more robust framework for understanding the systematics and evolutionary relationships within *Acmella*. The resemblance in sculptural pattern between *A. krueangensis* sp. nov. and the previously reported *Acmella* species suggests morphological continuity; however, molecular evidence supports the recognition of *A. krueangensis* sp. nov. as the same species.

The other new species also show clear differences in shell sculpture. *Acmella thamsingensis* sp. nov. exhibits a well-defined spiral ridge accompanied by radial sculpture prominent on the upper part of the whorl but fades towards the base. In contrast, *A. changphueakensis* sp. nov. and *A. kanchanaditensis* sp. nov. display prominent spiral sculptures but differ in the inter-row grooves thickness, depth, and spacing. Similarly, *A. changphueakensis* sp. nov. closely resembles *Acmella* sp. 2 reported by Suksai and Dumrongrojwattana (2022), with both studies involving specimens collected from the same locality. However, the earlier work was based solely on morphological examination, whereas the present study incorporates DNA barcoding at the *COI* gene to validate species distinctiveness.

Overall, integrating morphological and molecular data reinforces the distinctiveness of the four newly described *Acmella* species and highlights the high level of cryptic diversity within cave ecosystems. These findings emphasize the value of combining traditional morphological approaches with molecular tools in the taxonomic study of microgastropods, particularly those inhabiting specialized and understudied environments such as tropical caves. In particular, molecular analyses based on *COI* DNA barcoding and phylogenetic reconstruction support the morphological differentiation among the new species. Despite sharing similar shell characteristics typical of the genus *Acmella*, each species exhibits distinct sculptural patterns. This pattern of congruence between morphological and molecular evidence is consistent with previous studies of *Acmella* species from Borneo, such as *A. cyrtoglyphe* and *A. polita* (Hendriks 2020). However, the *COI* gene analysis grouped Thai specimens with *A. polita* and *A. cyrtoglyphe* from Borneo in a single unresolved clade, indicating insufficient resolution to clarify their evolutionary relationships. Reliance on mitochondrial *COI* alone may limit the ability to distinguish closely related *Acmella* lineages in these regions. This unresolved topology suggests that additional markers, including nuclear genes, and multilocus phylogenetic analyses are needed to better resolve species boundaries and evolutionary history across Thailand and Borneo. Despite these limitations, our results support the validity of the new species and highlight the evolutionary diversification of *Acmella* within cave habitats.

While this study significantly enhances the understanding of *Acmella* diversity in Southern Thailand, several limitations should be acknowledged. Sampling was limited to a few cave systems, and ecological data on habitat preferences, behavior, and life history traits remain insufficient. Furthermore, genetic analyses were based solely on mitochondrial *COI* sequences from a small number of specimens, which do not adequately capture intraspecific variation. Future research should expand to include broader geographic sampling across Thailand and neighboring countries, employ multilocus genetic approaches, and incorporate ecological investigations to elucidate better the evolutionary history, population structure, and conservation needs of these specialized cave-dwelling microsnails.

## ﻿Conclusion

This study describes the discovery of four new *Acmella* species from cave systems in Southern Thailand, expanding our knowledge of the genus and its diversity within the Assimineidae family. The species show unique morphological and ecological adaptations to aphotic cave environments. Morphological and *COI* DNA barcoding data provide important insights into their systematics and evolutionary relationships. However, further molecular and ecological research is needed to understand their genetic diversity, evolutionary history, and conservation status, with an emphasis on their biogeographic distribution.

## Supplementary Material

XML Treatment for Acmella

XML Treatment for Acmella krueangensis

XML Treatment for Acmella thamsingensis

XML Treatment for Acmella changphueakensis

XML Treatment for Acmella kanchanaditensis

## ﻿Additional information

### Use of AI

No use of AI was reported.

### Author ORCIDs

Supattra Poeaim https://orcid.org/0000-0003-1293-3820

### Data availability

The dataset supporting this study has been deposited in the Global Biodiversity Information Facility (GBIF) Pensoft Integrated Publishing Toolkit (IPT). It is available at: https://ipt.pensoft.net/manage/resource?r=acmella_new_species_thailand_2025.
